# Development of the Organisational Health Literacy Responsiveness (Org-HLR) self-assessment tool and process

**DOI:** 10.1186/s12913-018-3499-6

**Published:** 2018-09-06

**Authors:** Anita Trezona, Sarity Dodson, Richard H. Osborne

**Affiliations:** 1 Deakin University, Waurn Ponds, Geelong, Victoria 3216 Australia; 2 The Fred Hollows Foundation, Melbourne, Australia

**Keywords:** Health literacy, Health literacy responsiveness, Health systems, Access, Health service improvement, Self-assessment

## Abstract

**Background:**

The World Health Organization describes health literacy as a critical determinant of health and driver of citizen empowerment and health equity. Several studies have shown that health literacy is associated with a range of socioeconomic factors including educational attainment, financial position and ethnicity. The complexity of the health system influences how well a person is able to engage with information and services. Health organisations can empower the populations they serve and address inequity by ensuring they are health literacy responsive. The aim of this study was to develop the Organisational Health Literacy Responsiveness self-assessment tool (Org-HLR Tool), and an assessment process to support organisations with application of the tool.

**Methods:**

A co-design workshop with health and social service professionals was undertaken to inform the structure of the tool and assessment process. Participants critiqued existing self-assessment tools and discussed the likely utility of the data they generate. A review of widely used organisational performance assessment tools informed the structure and self-assessment process. The Organisational Health Literacy Responsiveness (Org-HLR) Framework (with seven domains/24 sub-domains) provided the structure for the assessment dimensions of the tool. The performance indicators were drawn from raw data collected during development of the Org-HLR Framework.

**Results:**

Twenty-two professionals participated in the workshop. Based on the feedback provided and a review of existing tools, a multi-stage, group-based assessment process for implementing the Org-HLR Tool was developed. The assessment process was divided into three parts; i) reflection; ii) self-rating; and iii) priority setting, each supported by a corresponding tool. The self-rating tool, consistent with the Org-HLR Framework, was divided into: External policy and funding environment; Leadership and culture; Systems, processes and policies; Access to services and programs; Community engagement and partnerships; Communication practices and standards; Workforce. Each of these had 1 to 5 sub-dimensions (24 in total), and 135 performance indicators.

**Conclusions:**

The Org-HLR Tool and assessment process were developed to address a gap in available tools to support organisations to assess their health literacy responsiveness, and prioritise and plan their quality improvement activities. The tool is currently in the field for further utility and acceptability testing.

**Electronic supplementary material:**

The online version of this article (10.1186/s12913-018-3499-6) contains supplementary material, which is available to authorized users.

## Background

Health literacy has been defined as “the cognitive and social skills which determine the motivation and ability of individuals to gain access to, understand and use information in ways which promote and maintain good health” [1]. Population studies conducted in the United States (U.S.), Canada, Europe and Australia suggest that limited functional health literacy is a public health challenge in these countries [2–5], and that people with low functional health literacy may have less knowledge about their health conditions and treatments, poorer overall health status, and higher rates of hospitalisation than the rest of the population [6–8]. Low functional health literacy may also impact an individual’s ability to participate in decision-making, follow care recommendations, implement health-promoting behaviours, and engage with preventative health services [2, 9, 10].

Studies have also shown a relationship between health literacy and a number of socioeconomic factors, including educational attainment, financial position and ethnicity [5, 11]. A recent Australian study reported that health literacy varies across population groups. In particular, people born overseas, people who speak a language other than English at home, people with limited formal education, and people with chronic conditions were more likely to have low health literacy [12]. However, it must be acknowledged that health literacy is content and context specific. That is, a person’s ability to access, use and understand information is greatly influenced by the context in which they are required to apply the information to make health-related decisions [11].

The World Health Organization recently acknowledged health literacy as a critical determinant of health and a key driver of citizen empowerment and health equity in the Shanghai Declaration, which establishes a mandate for developing, implementing and monitoring strategies for strengthening health literacy in all populations [13]. Such strategies must extend beyond a focus on the health literacy of individuals, to include strategies that reduce the demands health systems place on people, and minimise barriers to them accessing services and information. By ensuring they are health literacy responsive, health and social care organisations can empower the populations they serve.

Health literacy responsiveness has been defined as “the provision of services, programs and information in ways that promote equitable access and engagement, that meet the diverse health literacy needs and preferences of individuals, families and communities, and that support people to participate in decisions regarding their health and social wellbeing” [14]*.* Some authors have suggested that health literacy responsive organisations foster a culture that promotes equity and inclusiveness, demonstrate effective leadership and management, and ensure robust data collection, monitoring and communications systems and processes are in place. They also foster effective communication practices, and have a strong commitment to building the capability of their workforce, engaging meaningfully with the communities they serve, and partnering effectively with other health and social service organisations [15–18].

Health care organisations and health systems must continuously improve to ensure they are effective, efficient and responsive to the needs of populations [19]. Organisational self-assessments can be useful for improving organisational performance and effectiveness by supporting benchmarking and monitoring, guiding continuous quality improvement activities, encouraging collaboration and inclusive problem solving, and promoting self-reflection and organisational learning [20]. They are increasingly being utilised to guide organisational ‘diagnosis’ and needs identification, and facilitate goal setting and quality improvement planning [21–23]. Such tools generally consist of a set of performance categories, accompanied by a set of ‘best practice’ statements and examples that set a benchmark for performance assessment and planning [24].

While the need for organisations to better respond to the health literacy needs of populations has been acknowledged [15, 25–27], there are few self-assessment tools available to support organisations to assess their health literacy responsiveness. To date, most of the tools that have been developed for this purpose have been based on the ‘ten attributes of a health literate organisation’ proposed by the U.S. Institute of Medicine [15]. The ten attributes were derived through expert opinion and a synthesis of the literature on health literacy research and practice [15]. The ten attributes are framed as a set of goals, supported by a list of strategies or actions organisations can implement to improve their responsiveness. Four of the attributes relate to communication, in addition to one each on leadership, planning and evaluation, preparing the workforce, involving consumers, meeting the needs of populations, and ensuring easy access. These attributes have been incorporated into several organisational assessment or audit tools outside of the U.S. context, including the Enliven Organisational Health Literacy Self-Assessment Resource [28], the Health Literacy Review: A Guide [29], and the Vienna Health Literate Organizations Instrument (V-HLO-I) [30].

Self-assessment tools are more likely to be effective when they are appropriate in conceptual scope and assessment content, provide diagnostic guidance, and have high validity [31]. Organisational models and frameworks serve as the reference standard for self-assessment [31]. Thus, to inform the self-assessment tool developed in this study, we developed the Organisational Health Literacy Responsiveness (Org-HLR) Framework [14]. In summary, the Org-HLR Tool was developed using data generated from a series of concept mapping consultations with more than 200 professionals working in health and social services sectors. Concept mapping is a mixed methods process that incorporates nominal group techniques, unstructured sorting, multivariate statistical methods and in depth user input [32–34].

**Fig. 1:**
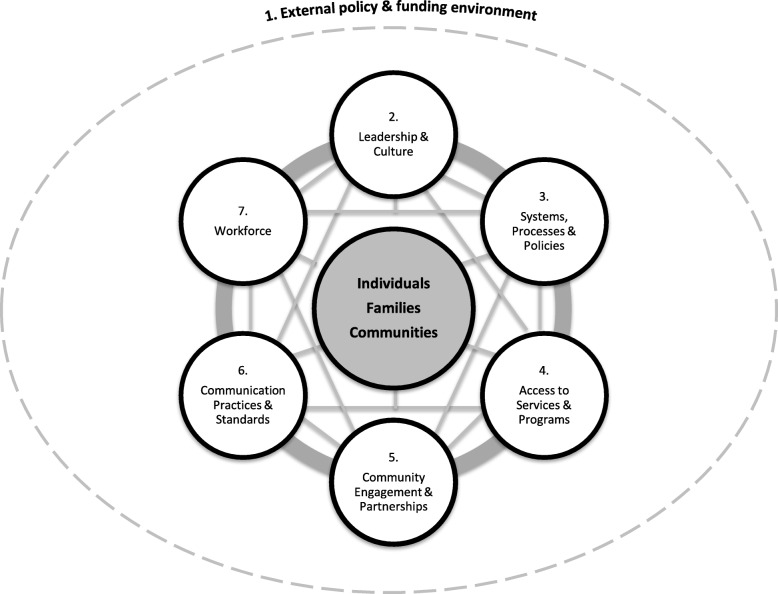
The Organisational Health Literacy Responsiveness (Org-HLR) Framework

Concept mapping has been widely used for over 25 years to aid the development of theory, plan for programs and social interventions, evaluate social programs, and develop measures and scales [35] including conceptual frameworks that have subsequently been found to be robust across settings [36–39]. It provides an inclusive and participatory approach and can be used to explore complex, multidimensional concepts and is therefore highly suited to assist with generation of the components of a health literacy responsive organisation. We further examined the concept mapping data using hierarchical cluster analysis to derive the domains and sub-domains of the Org-HLR Framework shown in Fig. 1, as well as the performance indicators that make up the self-assessment tool [14].

The aim of the current study was to develop the Organisational Health Literacy Self-Assessment Tool (Org-HLR Tool), and an assessment process to support organisations with the application of the tool.

## Methods

**Fig. 2:**
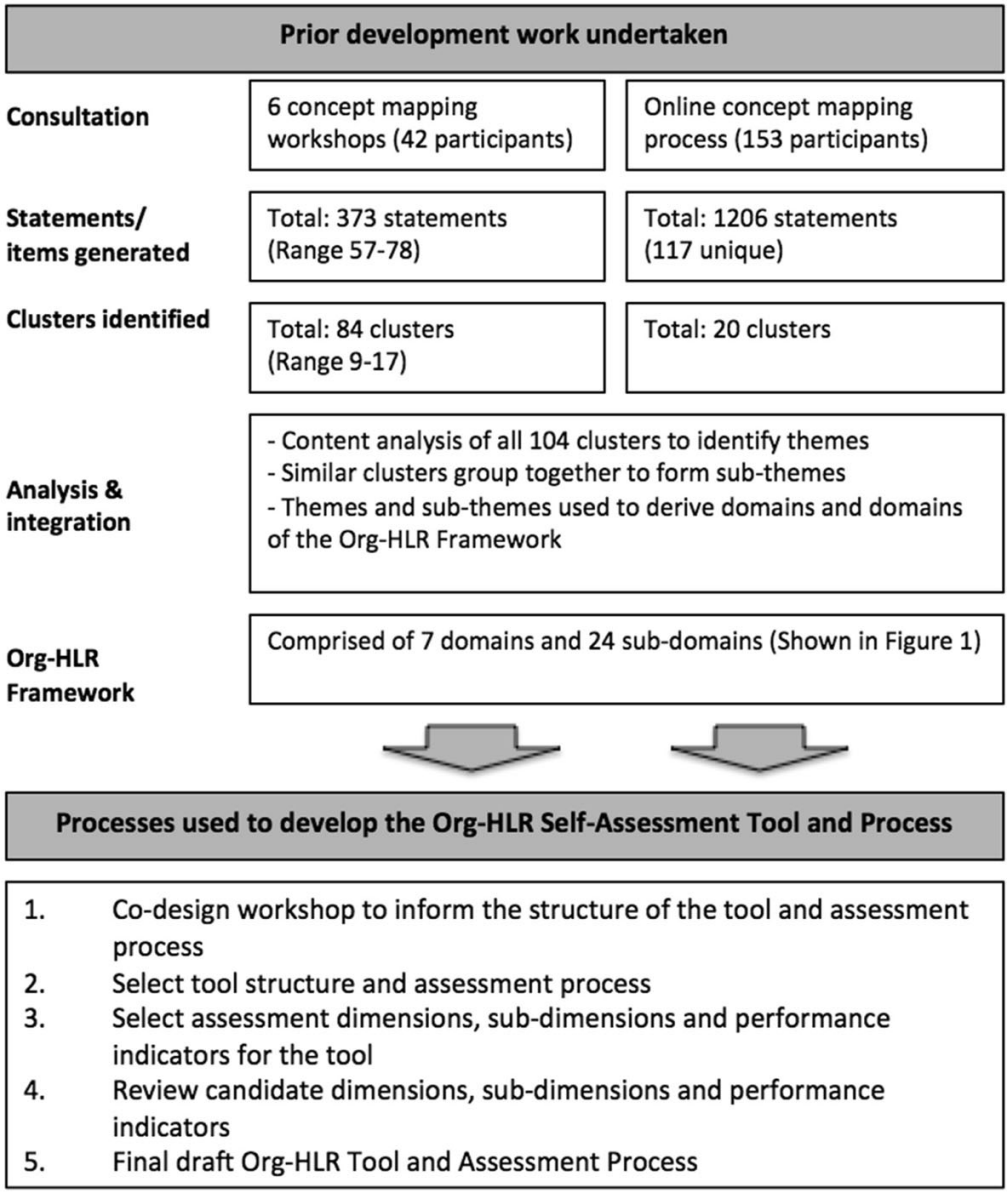
Steps undertaken in the development of the Org-HLR Self-Assessment Tool and assessment process

This study was undertaken in two parts. The first involved determining the structure of the self-assessment tool and an approach for undertaking the assessment process. The second involved selecting the assessment dimensions and performance indicators for the self-assessment tool. The activities and steps involved in this study are shown in Fig. 2. This study was approved by the Deakin University Human Research Ethics Committee (Study ID: 2012-295).

### Co-design workshop to inform the structure of the tool and assessment process

To inform the structure of the tool, and the approach for undertaking the assessment process, we conducted a co-design workshop with health and social service sector professionals. The aim of the workshop was to obtain information on the potentially useful characteristics of a self-assessment tool and assessment process from the perspective of practitioners and managers working within relevant organisational settings and contexts. An invitation to participate was distributed to the member organisations of four Primary Care Partnerships in a metropolitan region of Melbourne, Australia. Primary Care Partnerships are voluntary alliances of health and human service organisations, which work together to improve access to, and coordination of services [40]. A total of 22 professionals participated in the workshop who represented the full range of services in Victoria. They included representatives from hospitals (*n* = 5), community health services (*n* = 7), women’s health services (*n* = 2), local governments (*n* = 2), Primary Care Partnerships (*n* = 2), Primary Health Networks (*n* = 1) and other non-government organisations (*n* = 3). Participants were made up of people providing direct care to consumers, people managing staff who provide care, and people involved in delivering community based projects. They ranged from health promotion officers to executive managers of organisations. All participants provided written consent.

To commence the workshop, participants were introduced to the Org-HLR Framework, including a brief explanation of the domains and sub-domains. Presentation of the framework assisted to orient the group to the nature of the assessment being discussed. A group activity was then undertaken to obtain perspectives on the characteristics of a useful self-assessment tool and assessment process within their settings. This discussion focused particularly on potential ways a tool might be structured and administered rather than the content of the assessment itself. Participants were provided with four existing self-assessment tools and asked to critique them according to a set of guiding questions, which sought their views on the strengths and limitations of the tools, the potential barriers and enablers to implementing them, the types of data they generate and how useful that would be, and suggestions on ways the tools could be improved. The four tools used in this activity were: i) Assessing Chronic Illness Care (ACIC) Survey: V3.5 [41]; ii) Enliven Organisational Health Literacy Self-Assessment Resource [28]; iii) Health Literacy Review: A Guide [29]; and the Agency for Healthcare Research and Quality (AHRQ) Primary Care Health Literacy Assessment [42]. These tools were selected for their specific focus on health literacy or a related health system issue, and because they varied in their design, structure and mode of administration. The characteristics of these tools are described in Additional file 1. In a second activity, participants were asked to describe the characteristics of an ‘ideal’ self-assessment tool, based on their experience of undertaking assessments and planning improvement activities within their contexts. This second activity allowed the workshop participants to reflect upon the discussion of tool strengths and limitations and highlight to the research team those characteristics they considered particularly important.

### Determining the tool structure and assessment process

The feedback provided during the consultation workshop was analysed, using qualitative thematic analysis, to identify the perceived strengths and limitations of the example tools, the perceived utility of the data generated for supporting priority setting and action planning, and the perceived overall utility of the tools for assessing organisational performance in relation to health system issues. These key themes informed decisions regarding the structure of the Org-HLR Tool and assessment process.

We also reviewed a range of widely used organisational performance and self-assessment tools to identify their common characteristics, and to make judgements about their likely strengths and limitations, degree of implementation difficulty, and their potential to produce data that will support quality improvement planning, monitoring and evaluation. This included a review of factors such as clarity of the assessment dimensions, the length of the tools, the mode of administration, the rating systems and data collection methods used, and the types of instructions and guidelines provided to support implementation. We included two widely used organisational performance tools in the review, the Baldrige Criteria for Performance Excellence [43] and European Foundation for Quality Management (EFQM) Model [44], as well as more localised self-assessment tools relating to cultural competence [45–47], gender equity, [48] consumer-centred/coordinated care, [41, 49] and other tools. The useful elements of these tools were identified, adapted and incorporated into the structure of the Org-HLR Tool and assessment process.

### Selecting assessment dimensions, sub-dimensions and performance indicators for the tool

The seven domains of Org-HLR Framework (derived through prior development work described in Fig. 1) served as the broad assessment categories of organisational performance, while the sub-domains informed the sub-dimensions for each assessment dimension. The performance indicators were selected based on the statements generated through the concept mapping process utilised in the development of the Org-HLR Framework [14].

The statements generated through the concept mapping process were evaluated to determine a representative set of performance indicators (items) for each sub-dimension. In selecting each item within a dimension, consideration was given to ensuring good coverage, whether a wide range of professionals would be able to interpret and understand the item, whether they would be able to make a judgement about how well their organisation is addressing the item, and whether the item could realistically be operationalised into a concrete action to improve organisational practice and performance. In order to describe the breadth of the concept as expressed by participants during the concept mapping consultations, it was important to retain as many distinctions as possible whilst at the same time minimising the potential response burden associated with having too many assessment criteria. Duplicate statements were removed, conceptually similar statements combined, and a minimal set of candidate items for the assessment tool were selected through consensus by the research team.

### Step 4: Review of candidate dimensions, sub-dimensions and performance indicators for the tool

Independent researchers reviewed a draft version of the assessment tool comprising a proposed set of dimensions, sub-dimensions and performance indicators. Their task was to assess whether: i) the titles for each assessment dimension and sub-dimension provided appropriate coverage of the assessment topic; ii) titles appeared easy to interpret by a broad audience; and iii) performance indicators contained within each sub-dimension were both easy to interpret and represented the intended focus of the sub-dimension.

## Results

### Key themes derived from co-design workshop on the structure of the tool and assessment process

Participants in the co-design workshop reported the following to be important characteristics of a useful self-assessment tool: i) it has relevance and usefulness across a broad range of settings (i.e. community health, hospitals, primary care, local government; ii) it is capable of examining whole of organisation performance; iii) it is administered in a way that supports cross-team and cross-system conversations and action; iv) it generates quantitative and qualitative information that allows for identification of strengths and weaknesses; v) it is accompanied by instructions and guidelines as well as supporting resources and templates; vi) it is logically structured and divided into modules which are well defined and explained; and vii) it can be undertaken in a timely manner, without the support of external facilitators.

### Development of the assessment process

Based on the key themes derived from the co-design workshop and a review of existing self-assessment tools and processes, a multi-stage, group based assessment process for implementing the Org-HLR Tool was determined to be the most appropriate. This decision was based particularly on the view expressed by participants that the assessment process should facilitate conversations within the organisation and provide deep insights into the areas of strength and opportunities for development. Participants identified that the process of generating the assessment results was as important as the results themselves, in supporting change to occur within their organisations. Consequently, the assessment process was designed to be completed by organisations through a series of facilitated, multidisciplinary workshops. The proposed process was structured according to the Org-HLR Tool; i) reflection; ii) self-rating; and iii) priority setting.

#### Part 1: Reflection activity

The reflection activity, conducted over approximately 60–90 min, encourages reflection and discussion about health literacy concepts, the specific health literacy needs of clients and communities, and the organisation’s role in responding to them. It provides an opportunity to orientate members of the organisation to the assessment process and prepare them for the subsequent self-rating and priority setting tasks.

#### Part 2: Self-rating activity

The self-rating activity, conducted over approximately three to 4 h, enables organisations to assess their health literacy responsiveness against a set of performance criteria, as well as identify specific strengths and weaknesses in organisational capability and performance.

#### Part 3: Priority setting activity

The priority setting activity, conducted over approximately two to 3 h, supports organisations to prioritise actions and improvement activities based on the areas of weakness identified in the self-rating activity.

### Development of the structure and content of the self-assessment tool

The proposed self-assessment tool was divided into three parts, to correspond with the three assessment activities. This was complemented by a user-guide, which provided definitions, instructions and recommendations for undertaking the assessment process, as well as templates to support organisations to document the results and outputs of their assessment activities (User-Guide provided at Additional file 2).

#### Reflection tool

The reflection tool provided a set of questions for guiding reflection and group discussion about health literacy concepts and the specific health literacy needs of the organisation’s clients and communities. The proposed questions were:

i) How well do we currently understand the concepts of health literacy and health literacy responsiveness?
ii) How well do we currently understand the relationship between health literacy, health literacy responsiveness and consumer experiences and health outcomes?
iii) How well do we currently understand and promote equity, diversity and consumer-centred care?
iv) To what extent do we understand and acknowledge our role in making it easy for consumers and the broader community to access the information, programs and services we provide?
v) To what extent do we respond effectively to the needs of community members that experience barriers (for example due to financial circumstances, disability, mobility constraints, culture, language, low literacy, distance) to accessing support?

These questions were presented as a guide and organisations are encouraged to adapt the questions to their context.

#### Self-rating tool

The self-rating tool was divided into seven assessment dimensions, each of which is made up of 1 to 5 sub-dimensions (24 in total), and 135 performance indicators, as shown in Table 1.

**Table 1:** Assessment dimensions, sub-dimensions and number of performance indicators (PI) of the Org-HLR Tool

Assessment dimensions	Sub-dimensions	Number of Indicators
1. External policy and funding environment	1.1 External policy and funding environment	4
2. Leadership and culture	2.1 Financial management	3
	2.2 Leadership and commitment	4
	2.3 Health literacy is an organisational priority	4
	2.4 Equity and diversity focused	4
	2.5 Consumer-centred philosophy	3
3. Systems, processes and policies	3.1 Undertaking data collection and community needs identification	9
	3.2 Undertaking performance monitoring & evaluation	5
	3.3 Undertaking service planning & quality improvement	7
	3.4 Communication systems and processes	8
	3.5 Internal policies and procedures	6
4. Access to services and programs	4.1 Providing an appropriate service environment	3
	4.2 Supporting initial entry and ongoing access to services and programs	8
	4.3 Providing outreach services	3
5. Community engagement and partnerships	5.1 Undertaking community consultation and enabling consumer participation	8
	5.2 Partnerships with other organisations	6
6. Communication practices and standards	6.1 Communication principles/standards	10
	6.2 Providing health information	6
	6.3 Using media and technology	5
	6.4 Providing health education programs	3
7. Workforce	7.1 Recruiting and appropriate workforce	4
	7.2 Providing supportive working environments	3
	7.3 Providing practice tools and resources	8
	7.4 Providing ongoing professional development	11

In order to encourage discussion about organisational strengths and weaknesses, a global scoring system was derived. This scoring, applied at the sub-dimension level was regarded as more useful than at the individual performance indicator level. A truncated version of the self-rating tool is provided at Additional file 3, which shows two exemplar performance indicators for each sub-dimension of the tool. The descriptors for the five rating levels of the self-rating tool are described in Table 2.

**Table 2:** Scale descriptors for the Org-HLR Tool

Rating	Descriptor
1	There is no evidence that this occurs, and there is no support/commitment internally for undertaking work in this area.
2	There is no evidence that this occurs, but the organisation has made a commitment to it and planning has commenced.
3	There is evidence that this occurs sporadically across some parts of the organisation, but it is undertaken inconsistently and significant improvements are required.
4	There is evidence that this occurs consistently across most parts of the organisation, but improvements are required to embed it into organisational systems and processes.
5	This is routine practice, is undertaken consistently across all areas of the organisation, and has been embedded into organisational systems and processes.

A template was provided for recording the identified strengths and weaknesses at the end of each assessment dimension. The data recorded in the template is utilised to populate the priority setting tool template and prepare for/inform discussions during the priority setting stage of the assessment process.

#### Priority setting tool

The priority-setting tool provided a set of questions to guide a discussion on the organisation’s strengths and weaknesses. The proposed questions were:

I. What do we currently do well to support the health literacy needs of consumers and the community?
II. What could we do better to support the health literacy needs of consumers and the community?
III. What system/process/practice improvements need to occur within the organisation to strengthen our responsiveness to the health literacy needs of consumers and the community?
IV. Do we currently have the available expertise, capacity and system capability to implement the required improvements?

After discussing and rating organisational performance using the questionnaire, Participants are then prompted to generate a list of actions that need to be implemented to improve their performance against the seven assessment dimensions (based on the examples recorded during the self-rating activity). A template was provided for participants to rate the level of importance of the actions they identify and the level of resourcing required to implement them. The criteria for rating the priority level were:

1. This requires immediate action, as this is very likely to have significant impact on our overall performance, and prevents us from making improvements in many other areas (highest);
2. This requires action, as it may have a significant impact on our overall performance and prevents us from making improvements in some other areas; and
3. This requires gradual action, as it is not likely to have a significant impact on our overall performance and does not prevent us from making improvements in other areas (lowest).

The proposed criteria for recording the resourcing required are:

1. Can be achieved with existing resources;
2. Requires additional staff resources;
3. Requires additional financial resources; and
4. Requires additional staff and financial resources.

## Discussion

We sought to develop a self-assessment tool and process that enables organisations to identify their health literacy responsiveness strengths and limitations, and then prioritise and plan health literacy-related system improvement activities. We derived the Org-HLR Tool and process through co-design processes with a wide range of professionals working in the health and social services sectors, in order to ensure its relevance and utility within these sectors in Australia. Consequently, the Org-HLR Tool is comprised of seven assessment dimensions, 24 sub-dimensions (impact areas) and 135 performance indicators, and supported by a comprehensive assessment process, including reflection, self-rating and priority setting activities.

Ford & Evans [31] propose five key characteristics that influence the effectiveness and suitability of a self-assessment tool within a given context: i) conceptual domain; ii) concreteness; iii) diagnostic guidance; iv) affiliation (has been developed by a credible and respected institution); and v) validity. The development of the Org-HLR Tool ensured that it has strengths across each of these characteristics. Firstly, the tool was derived from a conceptual framework comprised of the elements of a health literacy responsive organisation, therefore its conceptual scope aligns specifically with the requisite assessment areas and overall objectives of a health literacy responsiveness self-assessment. The tool demonstrates concreteness insofar as it has a specific focus on a priori elements of a health literacy responsive organisation and uses concepts, language and terminology that reflect the context and experiences of the intended users. It is accompanied by instructions and templates that seek to increase the utility of the tool by managers and practitioners without the support of external experts or facilitators. The tool intends to provide sound diagnostic guidance by supporting the identification of organisational strengths and limitations, with an emphasis on “actionable improvement opportunities”. Finally, the Org-HLR Tool was developed using a participatory approach involving a wide range of health and social service organisations with clearly defined co-design methodology. All of the above elements support credibility and face validity with intended users.

The Org-HLR Tool is comprised of assessment dimensions and a comprehensive set of sub-dimensions that provide clear actionable areas for improving organisational performance. The empirical development of the assessment dimensions in collaboration with a wide range of professionals is likely to ensure it has validity and utility across a range of health and social service contexts, including community health, women’s health, hospitals, primary care services, local governments and other peak bodies and not-for-profit organisations.

The Org-HLR Tool differs from other health literacy self-assessment tools, in that it is incorporated into a comprehensive assessment process. We expect the proposed assessment process will maximise the utility and effectiveness of the Org-HLR Tool in a number of ways. Firstly, completion of the self-assessment process using group based workshops will enable participation by a diverse range of staff, thereby supporting cross-organisational learning, collaborative ‘diagnosis’ and inclusive decision-making [50]. This is likely to increase staff engagement in the improvement activities that flow from the self-assessment process. The use of qualitative and quantitative data collection methods will enable organisational benchmarking, and monitoring and evaluation of improvements over time [22, 51]. The priority-setting tool will encourage organisations to move beyond the problem identification stage, by incorporating the outputs of the assessment process into organisational planning and implementation activities [20]. Whilst a potential strength of the tool, its length and the requirement that multiple actors within an organisation participate in the process may limit its utility in some settings. Organisations considering undertaking the self-assessment may also find it necessary to orient key staff to the concepts of health literacy and health responsiveness in order to effectively engage them in the assessment process.

Although the Org-HLR Tool was developed for use by organisations in the Australian context, it is likely to have utility in healthcare settings in other countries. Health systems internationally share common features and are increasingly focused on ensuring effective engagement of the communities they delivery care to. It will however be necessary to adapt the tool for use in other contexts where barriers to access take different forms. The Org-HLR Tool and assessment process will need to be tested and validated in Australia, as in other countries to determine the applicability and comprehensibility of the assessment dimensions, and the feasibility of the assessment process. This is particularly important because each healthcare context may not only have a different mix of health services with different professional staff, but terminology to describe elements within services are likely to differ substantially. Healthcare systems that are fragmented, under-resourced or have complex insurance and gatekeeper structures, provide additional challenges to community members seeking care. The role of the Org-HLR Tool in these settings is yet to be tested and may require considerable modification.

## Conclusions

We developed the Org-HLR Tool and assessment process to address a gap in the availability and suitability of tools to support organisations to assess their health literacy responsiveness strengths and limitations, and prioritise and plan their quality improvement activities. The tool is likely to be relevant for a broad range of organisations across the health and social service sectors.

The tool is currently in the field for further testing of its utility and acceptability in a range of health and social service organisations across Victoria, Australia. This will allow us to identify the improvements and adaptations required to enhance its utility across these settings.

## Additional files

Additional file 1: Characteristics of tools used in the co-design workshop. (DOCX 96 kb)

Additional file 2: The Organisational Health Literacy Responsiveness (Org-HLR) Self-Assessment User Guide. (DOCX 425 kb)

Additional file 3: Org-HLR Self-Rating Tool (Truncated Version). (DOCX 42 kb)

## Abbreviations

ACIC: Assessing Chronic Illness Care
AHRQ: Agency for Healthcare Research and Quality
EFQM: European Foundation for Quality Management
Org-HLR: Organisational Health Literacy Responsiveness
PI: Performance Indicators
U.S: United States
V-HLO-I: Vienna Health Literate Organizations Instrument
