# Supplementary material for: Development of the Organisational Health Literacy Responsiveness (Org-HLR) self-assessment tool and process
Source: BMC Health Serv Res. 2018 Sep 6;18:694. doi: 10.1186/s12913-018-3499-6 (PMC6128002; doi:10.1186/s12913-018-3499-6)
Supplement: Supplementary file 1 — Characteristics of tools used in the co-design workshop. (DOCX 96 kb) [file 12913_2018_3499_MOESM1_ESM.docx]

**Additional File 1:**

**Characteristics of tools used in the co-design workshop**

| ***Tool*** | ***Purpose/aim*** | ***Context/Target*** | ***Structure & Format*** | ***Assessment Dimensions*** | ***Outputs & measures*** |
| --- | --- | --- | --- | --- | --- |
| Assessing Chronic Illness Care ACIC Survey: V3.5 | Identify improvement needs and guide quality improvement activities for chronic illness care. | Developed in the United States (US), for US health care organisations, namely primary care teams and medical practices | Length: 11 pages  Divided into eight assessment sections according to the Chronic Care Model dimensions with a corresponding set of sub-dimensions (N: 34). Is completed per physical site (i.e. hospital, practice) or team. Survey format, but mode of administration not stated (i.e. in groups or by individuals).  Instructions provided within the survey are limited to the rating system and interpretation. | - Organization of the healthcare delivery system - Community linkages - Practice level - Self-management support - Decision support - Delivery system design - Clinical information systems - Integration of chronic care model components | Provides quantitative outputs. Rating scale of 1-11, applied at the sub-dimension level and tallied to provide a total score and average score at the assessment dimension level. Scores at the assessment level are tallied to provide an overall score for chronic illness care. Descriptors for ratings according to a range; i.e. 0-2 = limited support. |
| Enliven Organisational Health Literacy Self-Assessment Resource | Identify the presence or absence of attributes of a health literate organisation. | Developed in Victoria, Australia based on the US Institute of Medicine’s Ten Attributes of a Health Literate Organisation. Adapted for use by Australian health care and social service organisations | Length: 34 pages  Divided into ten assessment sections, according to the ‘ten attributes’. Each section contains an information and resources page and a checklist of action statements (N: 85) under each attribute. Checklist format, completed by a single assessor. No instructions provided. | - Leadership - Planning, evaluation & quality improvement - Prepares the workforce - Includes populations in design, implementation & evaluation - Meets the needs of populations - Interpersonal communication - Access and navigation - Written information and communication - High-risk situations - Communicates about health plans | Provides quantitative and qualitative outputs. Uses a yes/no response option. Provides a column for marking whether an action statement is present under each attribute, and a column for documenting future actions and timelines etc. (qualitative data). |
| Health Literacy Review: A Guide |  | Developed in New Zealand for New Zealand-based organisations. The assessment dimensions adapted from the US Institute of Medicine’s Ten Attributes of a Health Literate Organisation. | Length: 68 pages  Provides a step-by-step guide on how to conduct a health literacy review (from data collection through to reporting and action planning) using the templates provided. A designated team completes review. Data collected through document reviews, observations and interviews. Data is analysed and presented according to the six assessment dimensions. | - Leadership & management - Consumer involvement - Workforce - Meeting the needs of the population - Access and navigation - Communication | Provides qualitative and quantitative outputs. Data recorded in multiple templates to (i.e. notes on document reviews, observations of interactions, interview discussions). Staff survey templates provide 4 and 6-point response options. Environment observation template provides a checklist. A checklist template is also provided for guiding analysis of the six dimensions. |
| Agency for Healthcare Research and Quality (AHRQ) Primary Care Health Literacy Assessment | Identify health literacy-related improvement needs to inform a health literacy improvement plan. | Developed in the US as part of the AHRQ Health Literacy Universal Precautions Toolkit (2^nd^ edn), comprising a suite of tools and resources to support US-based primary care practices to address health literacy. | Length: 7 pages  Divided into five assessment sections, with a corresponding set of indicators (N: 51). Survey format, can be completed by multiple staff. Group discussions about results of survey encouraged. No instructions provided within survey, but brief instructions included in toolkit. | - Prepare for practice change - Improve spoken communication - Improve written communication - Improve self-management & empowerment - Improve supportive systems | Provides quantitative outputs. Uses a four-point response option (doing well, needs improvement, not doing and not sure/NA), applied to each indictor. |
