# Supplementary material for: Development of the Organisational Health Literacy Responsiveness (Org-HLR) self-assessment tool and process
Source: BMC Health Serv Res. 2018 Sep 6;18:694. doi: 10.1186/s12913-018-3499-6 (PMC6128002; doi:10.1186/s12913-018-3499-6)
Supplement: Supplementary file 2 — The Organisational Health Literacy Responsiveness (Org-HLR) Self-Assessment User Guide. (DOCX 425 kb) [file 12913_2018_3499_MOESM2_ESM.docx]

**
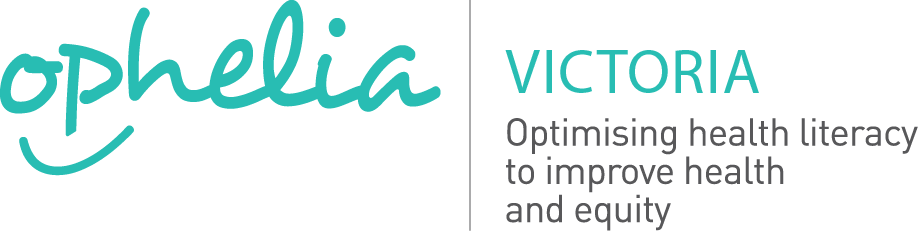
**
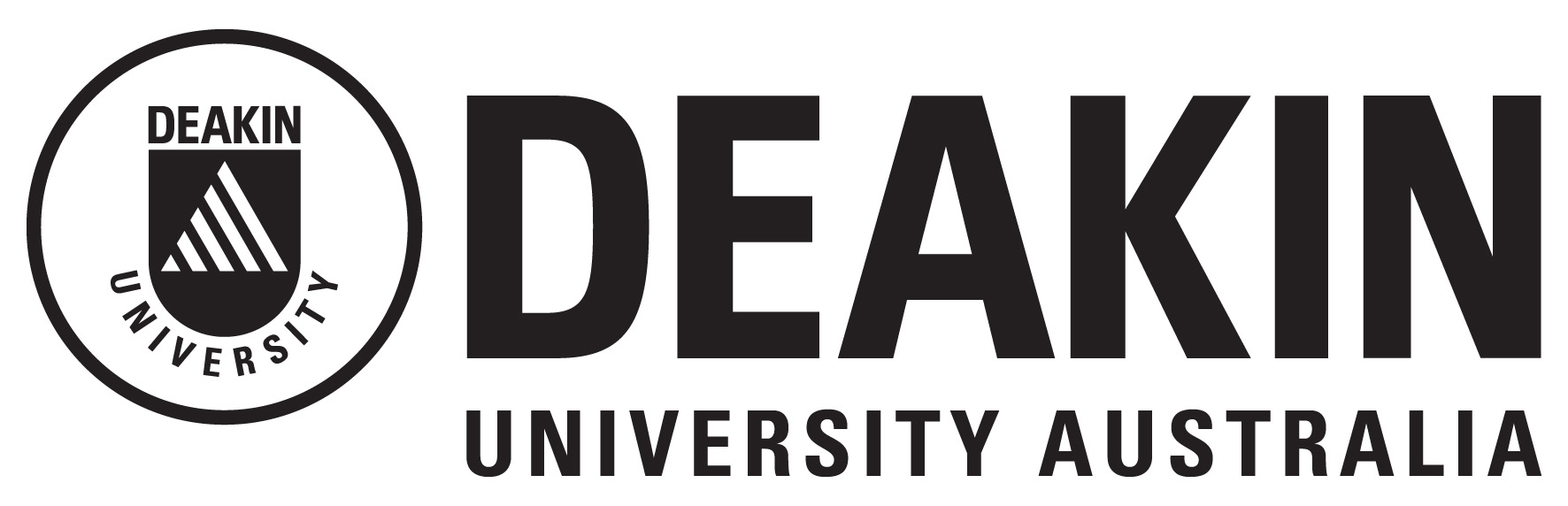


**The Organisational Health Literacy Responsiveness (Org-HLR)**

**Self-Assessment Guide**

***Pilot Version 1 – June 2016***

**Authors**

**Ms Anita Trezona**

**Dr Sarity Dodson**

**Professor Richard H Osborne**

**Acknowledgements**

**Authors**

Ms Anita Trezona, Dr Sarity Dodson and Professor Richard H Osborne

Health Systems Improvement Unit, Centre for Population Health Research, School of Health and Social Development, Deakin University.

**Contributors**

Mr Roy Batterham, Dr Alison Beauchamp and Professor Gerald Elsworth

Health Systems Improvement Unit, Centre for Population Health Research, School of Health and Social Development, Deakin University.

**Project Partners**

The Org-HLR Self-Assessment Tool and Guide were developed within the Ophelia (OPtimising HEalth LItercy and Access) Project at Deakin University. The project is funded by an Australian Research Council (ARC) Linkage Project grant, with partnership funding from the Victorian Department of Health, Deakin University and Monash University.

**
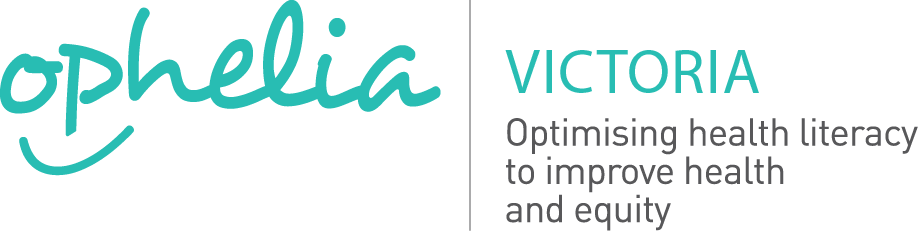
**


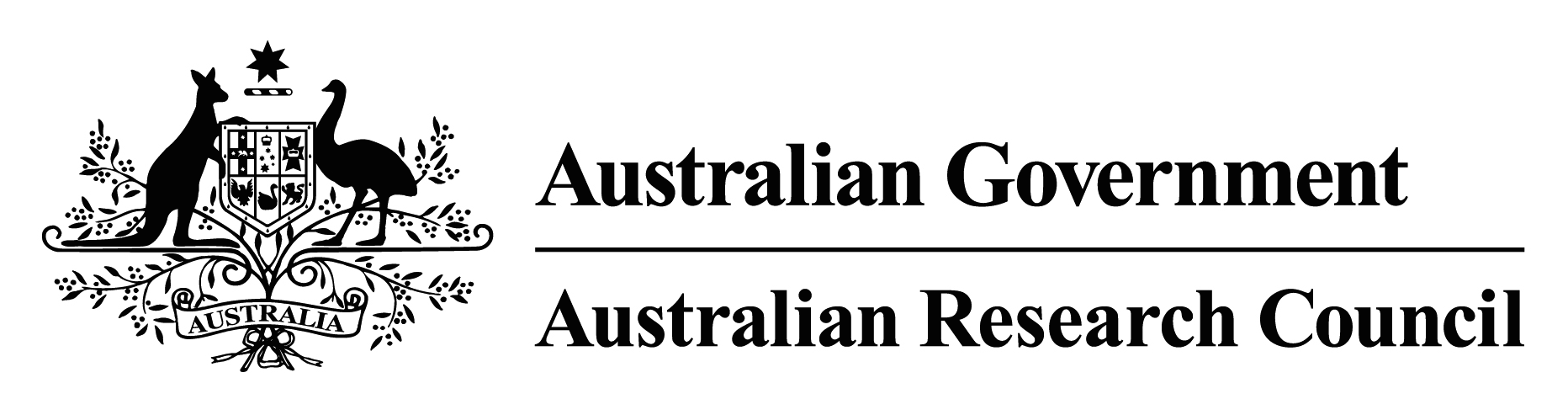


#
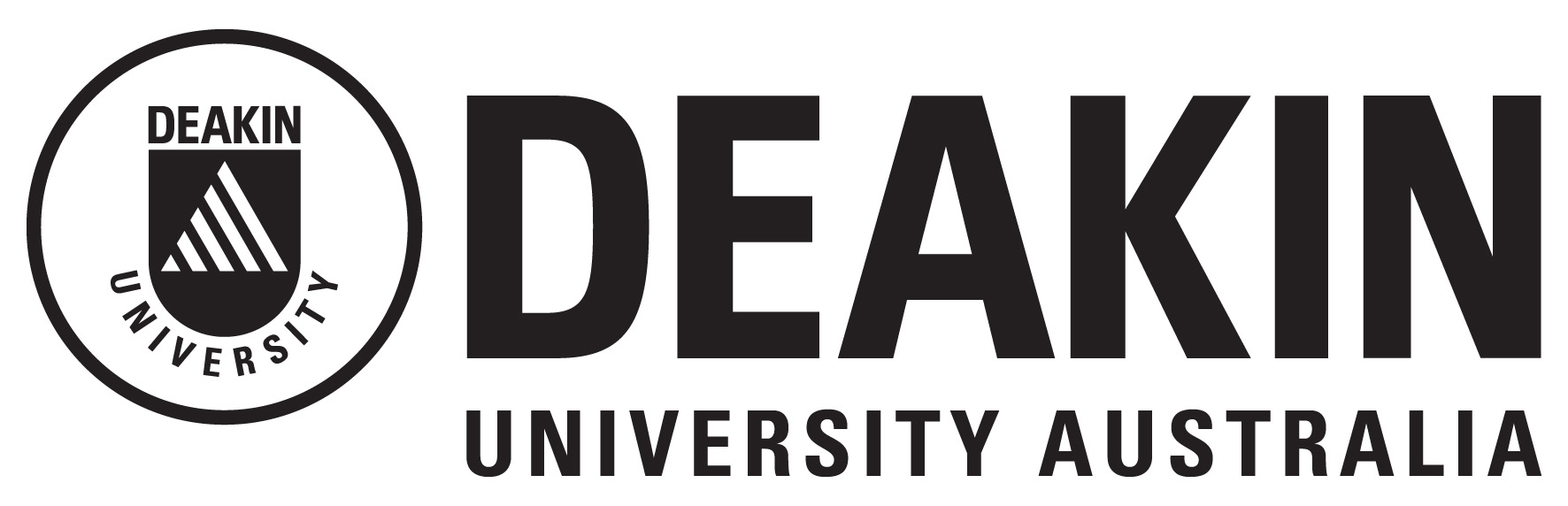


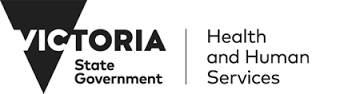


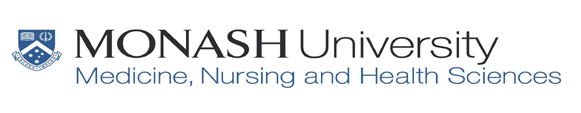


Table of Contents

Section One: Introduction to health literacy and health literacy responsiveness 4

What is health literacy? 4

What is health literacy responsiveness and why is it important? 4

Key concepts and terminology 5

Acronyms 7

Section Two: Orientation to the Organisational Health Literacy Responsiveness Self-Assessment Guide 8

The Org-HLR Self-Assessment Guide 8

The Org-HLR Tool 8

How the Org-HLR Tool was developed? 9

Who should use the Org-HLR Assessment Guide? 9

Section Three: Step-by-step guide to undertaking the Org-HLR Assessment 11

Step 1: Confirm organisational commitment to undertaking the assessment 11

Step 2: Determine your approach to the assessment 11

Step 3: Brief staff about the assessment process 12

Step 4: Undertake the assessment using the Org-HLR Tool 12

Step 5: Reporting the results of your assessment 14

Step 6: Develop your Health Literacy Action Plan 14

Step 7: Monitoring and follow up reviews 14

Section Four: The Org-HLR Tool and Templates 15

Reflection Tool 15

Self-Rating Tool 16

Priority Setting Tool 31

Health Literacy Action Plan Template 36

References 38

# Section One: Introduction to health literacy and health literacy responsiveness

## What is health literacy?

Health literacy is a multidimensional concept, comprising a range of cognitive, personal and social abilities and attributes at the individual level, as well as a broader range of contextual, socio-cultural and societal level factors ([Batterham et al., 2014](#_ENREF_1); [Sorensen et al., 2012](#_ENREF_6)).

Health literacy refers to the “personal characteristics and social resources needed for individuals and communities to access, understand, appraise and use information and services to make decisions about health, or that have implications for health. Health literacy includes the capacity to communicate, assert and enact these decisions” ([Dodson et al., 2014](#_ENREF_3)). Health literacy influences, and is influenced by, the health behaviours and circumstances of individuals and communities, as well as the characteristics of social, political and health systems.

That is, while individuals and communities have health literacy strengths and limitations that influence their ability to engage with information and services, health and social systems also have strengths and limitations in the degree to which they respond to the health literacy needs of the people they serve.

## What is health literacy responsiveness and why is it important?

Health literacy responsiveness refers to the capacity of organisations to ensure that programs, services and information are available and accessible to individuals and communities with varying health literacy strengths and limitations ([Dodson et al., 2014](#_ENREF_3)).

Health literacy is an important public health issue and it is now well recognised that government, health, social and community service organisations have a responsibility to respond appropriately and effectively to the health literacy needs of their clients and communities. Specifically, organisations have an important role to play in ensuring that their services, programs and information are available and accessible to everyone.

A number of studies have shown that people with limited health literacy experience poorer health outcomes, including less knowledge about their health conditions and treatment regimes, poorer overall health status, poorer self-reported physical and mental health, and higher rates of hospitalisation than the rest of the population ([Parker et al., 2003](#_ENREF_5); [Wolf et al., 2005](#_ENREF_7)). They also have decreased ability to share in decision-making about their health, less knowledge of health promoting behaviours, and are less likely to seek preventative health services ([Ishikawa and Yano, 2008](#_ENREF_4)).

A recent study revealed that health literacy varies significantly across population groups in Victoria. In particular, it found that people born overseas, people who speak a language other than English at home, and people with limited formal education are more likely to have low health literacy. It also revealed that women and people with multiple chronic conditions were vulnerable to lower health literacy ([Beauchamp et al., 2015](#_ENREF_2)). These findings highlight the importance of organisations having a strong understanding of the specific health literacy needs of the population groups within their community, in order to develop and deliver programs, services and information in ways that address their specific needs.

By increasing their health literacy responsiveness, organisations improve their ability to support individuals and communities to seek out and fully engage with their services, programs and information, as well as actively participate in, influence, and make informed decisions about their health and wellbeing. This is likely to significantly improve health outcomes and increase health equity for the people and communities they serve.

## Key concepts and terminology

**Access**

Access in the health context means individuals and communities have the right and opportunity to access programs, services and information when and where they need them. This includes gaining initial entry into the health system, and maintaining full engagement with all relevant services for as long as they are required. Access requires that health systems and services are approachable, available, acceptable, affordable, and appropriate, based on the needs and preferences of individuals and communities.

**Consumer-centred care**

Patient-centred care is health care that is respectful of, and responsive to, the preferences, needs and values of patients and consumers. It includes respecting the needs and preferences of consumers, treating consumers with dignity, providing emotional support and physical comfort, communicating effectively, coordinating care, involving family and carers, sharing decision-making and ensuring access to care.

*Related terms include;* client-centred care, person-centred care, consumer engagement

**Community engagement**

Community engagement is a process of working collaboratively with members of a community (can be defined by demographics, geographic location or shared interest) in the decision-making, planning, implementation, evaluation and governance of services, programs and initiatives. This involves building authentic partnerships and mutual respect, enabling inclusive participation, and power sharing for mutual benefit.

*Related terms include;* community participation, community mobilisation, community empowerment, community development, community partnerships, citizen engagement, citizen participation, citizen empowerment.

**Consumer participation**

Consumer participation is the right of consumers to be informed about and meaningfully involved in decision-making about their health care and treatment. This includes being involved in their own health care, having input into the way health services operate, and informing health policy, planning and service delivery.

**Cultural competence**

Cultural competence is a set of values and principles, attitudes, beliefs and behaviours, policies and structures that enable organisations and practitioners to work effectively cross-culturally. A culturally competent organisation is one that values diversity, assesses its awareness of culturally diverse communities, as well its assumptions an biases, has the ability to acquire cultural knowledge, is responsive to the specific needs of culturally diverse groups, involves culturally diverse groups in service and program planning, delivery and evaluation, and systematically incorporates each of these factors into the way it is structured and operates.

**Diversity**

Diversity means recognising and respecting the differences between people from all backgrounds, as well as acknowledging the unique knowledge, skills and experiences they contribute to communities and societies as a whole. Diversity means being inclusive and supportive of people with differences in culture, ethnicity, Aboriginality, sexual orientation, gender identity, age, socioeconomic status, geographic location, and abilities, and ensuring their full participation in all aspects of life.

**Gender diverse**

Gender refers to the socially constructed characteristics of women and men, such as norms, roles and relationships of, and between groups of women and men. Gender is a complex interrelationship between a person’s sex (biology), their internal sense of self as male, female, both or neither (gender identity) and their presentations and behaviors (gender expression) related to that identity, including their gender role. Gender diverse or gender diversity refers to the spectrum of gender identities that do not conform to the binary definition of gender as male and female, and the constructed gender roles assigned to them.

*Related terms include;* gender fluid, gender expansive

**Health education**
Health education involves structured opportunities for learning using forms of communication designed to improve health literacy, including improving knowledge, and developing life skills which are supportive of individual and community health. It includes the communication of information concerning the underlying social, economic and environmental conditions impacting on health, as well as individual risk factors and risk behaviours, and use of the healthcare system.

**Health equity**

Health equity means all people have an equal opportunity to develop and maintain their health, through the fair and just distribution of, and access to resources for health, including social and economic resources.

**Health inequities**

Health inequities are health differences which are systematic, and produced by social norms, policies, and practices that tolerate or actually promote the unfair distribution of, and access to power, wealth, and other necessary social resources.

**Social Determinants of health**

Social determinants are the structural factors and conditions of daily life that impact on health and wellbeing, including the distribution of power, income, goods and services, as well as the circumstances of peoples lives, such as their access to health care, schools and education; their conditions of work and leisure; their homes, communities, and rural or urban settings.

## Acronyms

CALD Culturally and Linguistically Diverse

QLBTIQ Gay, Lesbian, Bisexual, Transgender, Intersex, Queer

# Section Two: Orientation to the Organisational Health Literacy Responsiveness Self-Assessment Guide

## The Org-HLR Self-Assessment Guide

The Organisational Health Literacy Responsiveness Self-Assessment Guide aims to support organisations to assess their health literacy responsiveness, identify their health literacy strengths and limitations, and prioritise their health literacy improvement activities. The guide includes information on preparing for and communicating with staff about the assessment process, using the self-assessment tools, reporting the results of the assessment, developing a health literacy action plan and monitoring improvements over time. Section Three provides a step-by-step guide to undertaking the Org-HLR Assessment, and Section Four provides the tools and templates to guide the assessment process.

## The Org-HLR Tool

The Org-HLR Tool has been designed to bring staff from all levels of an organisation together to share perspectives and make judgements about how well the organisation supports clients and the broader community to fully access and engage with its services and programs. By encouraging open communication and discussion between staff, it has the potential to facilitate deep insights into aspects of organisational capacity and performance. The Org-HLR Tool is divided into three parts; i) reflection tool; ii) self-rating tool; and iii) priority setting tool.

***Reflection Tool:*** Aims to encourage reflection and open-ended discussion about health literacy concepts, the specific health literacy needs of clients and communities, and the role of organisations in responding to these health literacy needs.

***Self-Rating Tool:*** Aims to assess the health literacy responsiveness of organisations against a set of criteria and performance indicators.

***Priority Setting Tool***: Aims to support organisations to identify their health literacy improvement needs, and determine priorities for implementing health literacy improvement activities.

## How the Org-HLR Tool was developed?

The Org-HLR Tool was developed following a series of consultations involving more than 200 professionals working in the health and social services sectors across Victoria and Australia. During these consultations, participants shared their knowledge and perspectives on the ways in which organisations can better support and respond to the health literacy needs of their clients and communities.

Using quantitative and qualitative methods, the Organisational Health Literacy Responsiveness Framework was developed (see Table 1 below). This framework comprises seven core domains and describes the characteristics, values and actions of health literacy responsive organisations. The Organisational Health Literacy Responsiveness Framework then informed the structure, domains, impact areas and performance indicators of the Org-HLR Self-Rating Tool. A review of existing self-assessment tools as well as a targeted consultation involving professionals working in the health and social services sectors were undertaken to inform the mode of administration, sequence of activities and reporting format to accompany and support the Org-HLR Tool.

## Who should use the Org-HLR Assessment Guide?

The Org-HLR Tool is suitable and recommended for use by all organisations responsible for providing health and health related information, services and programs to individuals and communities, including:

- Community health services
- Women’s health services
- Aboriginal Community Controlled Organisations
- Mental health services
- Primary health care services
- Hospitals
- Drug and alcohol services
- Maternal and child health services
- Community and social service organisations
- Primary Care Partnerships
- Peak bodies and specialist organisations
- Local, State and Commonwealth Government organisations

**Table 1: Domains and Sub-Domains of the Organisational Health Literacy Responsiveness Framework**

| **Domains** | **Sub-Domains** |
| --- | --- |
| 1. Policy and funding mandate | - Policies, frameworks, standards, funding, incentives |
| 2. Leadership and culture | - Financial management - Leadership and commitment - Health literacy is an organisational priority - Equity and diversity focused - Consumer-centred philosophy |
| 3. Systems, processes and policies | - Data collection and community needs identification - Performance monitoring and evaluation - Service planning and quality improvement - Communication systems and processes - Internal policies and procedures |
| 4. Access to programs and services | - Service environment - Initial entry and ongoing access - Outreach services |
| 5. Community engagement and partnerships | - Community consultation and ensuring consumer participation - Partnerships with other organisations |
| 6. Communication with consumers | - Communication principles/standards - Health information provision - Use of media and technology - Health education programs |
| 7. Workforce | - Recruitment - Supportive working environments - Practice tools and resources - Ongoing professional development |

# Section Three: Step-by-step guide to undertaking the Org-HLR Assessment

## Step 1: Confirm organisational commitment to undertaking the assessment

Before commencing the assessment, it is important that you gain the authorisation of your senior managers to undertake the process. It is also important that a manager or leadership group leads the assessment process. Their commitment is critical for ensuring the assessment is adequately positioned as a priority for your organisation, that there are resources available to support the assessment process, and that ultimately there is a commitment to implementing the required practice and service improvements identified through the assessment process.

## Step 2: Determine your approach to the assessment

While it is important that a manager or leadership group leads the assessment process, the second step in the assessment process is to determine your approach. Some of the factors to consider are:

- Who is the most appropriate manager to act as the project sponsor/coordinator?
- Do you need to establish a working group or committee to coordinate assessment activities?
- Which staff should be involved, and how many?
- Will you complete each part of the assessment with one group of participants, or complete the assessment within teams/disciplines/departments etc.
- Will you engage an external facilitator to guide the assessment process?
- How will the results of the assessment be used, and where will they be reported?

In order to gain a comprehensive overview of your performance, it is recommended that staff from all areas of your organisation are involved in the assessment process, including executives, managers, practitioners, administrative staff and consumer representatives. If you are a small organisation, it may be appropriate to include all staff in the assessment process.

## Step 3: Brief staff about the assessment process

Once you have decided on your approach to the assessment, it is recommended that you meet with the staff that are going be involved to provide them with information on the purpose of the assessment, how it will be undertaken, how they will be involved and the anticipated timelines.

It is important that all relevant staff fully understand the purpose of the assessment and how the outcomes of the process will inform planning and quality improvement activities across the organisation.

## Step 4: Undertake the assessment using the Org-HLR Tool

The Org-HLR Tool is divided into three parts; i) reflection; ii) self-rating; and iii) priority setting, each of which is completed through a facilitated workshop. The assessment process may be undertaken with one group of staff from across your whole organisation, or it may be undertaken with multiple groups (at the discipline/team/department level) simultaneously.

If you decide to undertake the assessment with multiple groups, you will need to synthesise the data from each group’s assessment into one report in order to develop your whole of organisation health literacy action plan.

***Reflection on health literacy concepts and client/community health literacy needs***: The first part of the Org-HLR Tool takes approximately 60-90 minutes to complete, and encourages reflection and open-ended discussion among staff about health literacy concepts, the specific health literacy needs of your clients and communities, and your organisation’s role in responding to these health literacy needs. This part of the assessment also provides an opportunity to reiterate the purpose of the assessment process, and prepare staff for the self-rating and priority setting components of the assessment. *A* set of guiding questions is provided, however these can be modified to suit the specific needs and context of your organisation.

It is recommended that you document the discussions of this workshop, as the information provided by staff may be useful for informing aspects of organisational plans, processes or policies at a later date.

***Self-rating:*** The second part of the Org-HLR Tool is critical, as it provides an opportunity for you to assess your organisation’s health literacy responsiveness against a set of criteria. This component of the assessment generally takes 3 to 4 hours to complete. The self-rating tool is divided into a set of impact areas and performance indicators for each of the following health literacy responsiveness domains:

1. Policy and funding mandate

2. Leadership and culture

3. Systems, processes and policies

4. Access to programs and services

5. Community engagement and partnerships

6. Communication with consumers

7. Workforce

For this part of the assessment, you are encouraged to have an open discussion within your workshop group about how well your organisation is performing against each of the performance indicators (using examples/evidence where possible), before reaching a consensus view about the appropriate overall rating for each impact area. You are also encouraged to document examples of things you are doing well, and things you need to improve in order to be more health literacy responsive. The information you provide here will assist you with the priority setting activities in part three of the assessment.

Depending on the availability of staff time, you may choose to undertake this part of the assessment over multiple sessions. However, it is important to ensure that the same staff participate in each session to maintain consistent input throughout the assessment process.

***Priority setting***: The final part of the assessment aims to support your organisation to identify aspects of your systems, processes and practices that require improvement, and then determine priorities for implementing health literacy improvement activities. This part of the assessment usually takes 2-3 hours to complete, and starts with a brief reflection and discussion on your organisation’s strengths and weaknesses. You are then encouraged to document the actions required to improve your responsiveness, rate the level of importance of each action, and identify the resources required to implement them.

## Step 5: Reporting the results of your assessment

It is important to determine your audience for reporting the assessment results, and the way in which they will be reported. At a minimum, it is recommended that you have a mechanism for reporting the results to managers within the organisation, as they will be required to lead the health literacy planning process and implementation of quality improvement activities. For some organisations, it may be appropriate to report the results to a health literacy working group, quality improvement committee, management/executive meetings or even the Board.

If you decide to undertake your assessment with multiple groups, it is recommended that you collate the results into one report so that managers and participants in the assessment are provided with a comprehensive overview of your organisation’s performance.

You may also encourage your managers to share the results of the assessment with staff in their teams, to raise their awareness of the organisational factors that influence health literacy, as well as how well your organisation is responding to the health literacy needs of your clients and communities. It may also help to prepare staff for quality improvements activities and organisational changes that impact directly on their work.

## Step 6: Develop your Health Literacy Action Plan

There is an Action Plan Template provided in Section Four to assist you with the development of a whole-of-organisation health literacy action plan. In order to ensure the action plan is effectively implemented across the organisation, that adequate resources are allocated to improvement activities, and actions/strategies are incorporated into other relevant organisational plans and processes, it is recommended that a manager or leadership group completes or leads the action planning process.

## Step 7: Monitoring and follow up reviews

It is recommended that you undertake follow up assessments annually, as a way of monitoring your organisational performance over time and evaluating the impact of your health literacy improvement activities.

For follow up reviews, you may find it more practical to complete part two of the assessment only, particularly if you have developed a health literacy action plan that spans multiple years and you are therefore not seeking to establish new priorities.

# Section Four: The Org-HLR Tool and Templates

## Reflection Tool

| **Date** |  |
| --- | --- |
| **Facilitator** |  |
| **Note Taker** |  |
| **Participants** |  |

| **Purpose** | The purpose of this tool is to enable reflection and open-ended discussion about health literacy concepts, the specific health literacy needs of your clients and communities, and your organisation’s role in responding to these health literacy needs. This is an opportunity to seek a range of views and perspectives from staff, and all participants in the assessment group are encouraged to contribute their views. |
| --- | --- |
| **Questions** | 1. How well do we currently understand the concepts of health literacy/health literacy responsiveness?  2. How well do we currently understand the relationship between health literacy/health literacy responsiveness and consumer experiences/health outcomes?  3. How well do we currently understand and promote equity, diversity and consumer-centred care?  4. To what extent do we understand and acknowledge our role in making it easy for consumers and the broader community to access the information, programs and services we provide?  5. To what extent do we respond effectively to the needs of community members that experience barriers (for example due to financial circumstances, disability, mobility constraints, culture, language, low literacy, distance) to accessing support? |

**Org-HLR Self-Rating Tool (Truncated Version)**

| **1. Policy and funding mandate** |
| --- |
| **Performance indicators** |
| 1.1. There are policy frameworks available to guide health literacy work within our organisation |
| 1.4. We are provided with incentives to drive health literacy responsive practice |

| **2. Leadership and culture** |
| --- |
| **Impact area and performance indicators** |
| **2.1. Financial management** |
| 2.1.1. Our organisation allocates adequate financial resources to appropriate service and program delivery |
| 2.1.3. Health literacy improvement activities are resourced over the long term |
| **2.2. Leadership and commitment** |
| 2.2.1. Our organisation has a vision and mission that explicitly expresses our commitment to equity, diversity and consumer-centred care |
| 2.2.4. There are health literacy champions within our organisation who advocate for positive change and improvements |
| **2.3. Health literacy is an organisational priority** |
| 2.3.2. Our organisation has clearly defined health literacy goals and objectives |
| 2.3.3. Health literacy is included in our strategic plan/business/operational plans |
| **2.4. Equity and diversity focused** |
| 2.4.1. Our organisation celebrates diversity and promotes acceptance and inclusion of people from all diverse groups |
| 2.4.4. Equity and diversity principles are embedded into organisational plans and policies |
| **2.5. Consumer-centred philosophy** |
| 2.5.1. Health literacy is viewed as a an individual and community asset and right |
| 2.5.2. There is a commitment to delivering consumer-centred care at all levels of our organisation |

| **3. Systems, processes and policies** |
| --- |
| **Impact area and performance indicators** |
| **3.1. Undertaking data collection and community needs identification** |
| 3.1.3. Comprehensive community needs assessments are regularly and systematically undertaken to develop a knowledge of community demographics, determine barriers to access and inform service and program |
| 3.1.9. Data are disaggregated according to gender, culture and language |
| **3.2. Undertaking performance monitoring and evaluation** |
| 3.2.1. A set of health literacy standards have been established within our organisation |
| 3.2.3. There is a process in place for monitoring, evaluating and reporting on the quality of our health literacy practice |
| **3.3. Undertaking service planning and quality improvement** |
| 3.3.1. We have a systematic process in place for undertaking service and program planning |
| 3.3.5 There is a mechanism in place for ensuring the outcomes of quality improvement activities are documented, reported and incorporated into future planning processes |
| **3.4. Communication systems and processes** |
| 3.4.4. Recall and reminder systems are in place to support appointment confirmation and post-appointment follow up |
| 3.4.6. There is a system and process in place for developing and reviewing all communication materials to ensure they meet health literacy standards |
| **3.5. Internal policies and procedures** |
| 3.5.1. We have policies and procedures in place to support effective engagement with clients and the community |
| 3.5.5. Relevant staff are involved in the development and review of policies and procedures |

| **4. Access to programs and services** |
| --- |
| **Impact area and performance indicators** |
| **4.1. Providing an appropriate service environment** |
| 4.1.1. Our buildings and venues/facilities are accessible (e.g. affordable parking, ramp access, and close to public transport) |
| 4.1.2. Our services provide a welcoming and supportive environment for diverse and vulnerable groups (including for Aboriginal people, CALD communities, GLBTIQ communities etc.) |
| **4.2. Supporting initial entry and ongoing access to services and programs** |
| 4.2.1. We have clear access and referral pathways in place |
| 4.2.8. Clients are provided with resources that support them to navigate their way through the health system |
| **4.3. Providing outreach services** |
| 4.3.1. We utilise a range of service delivery models to ensure we engage people and communities who are unable to attend services in person (e.g. outreach services, out of ours services, mobile and online) |
| 4.3.3. We deliver community based events to provide information and services to people not engaged in services (e.g. screening and health checks, immunisation) |

| **5. Community engagement and partnerships** |
| --- |
| **Impact area and performance indicators** |
| **5.1. Undertaking community consultation and enabling consumer participation** |
| 5.1.1. Our organisation consults with the community to develop an understanding of their health and health literacy needs |
| 5.1.5. We engage clients and communities in all aspects of service and program planning |
| **5.2. Partnerships with other organisations** |
| 5.2.1. Our organisation works collaboratively with service partners to co-design services, programs, materials and referral pathways |
| 5.2.4. Our organisation engages with services outside the health sector, such as housing, education and employment, to ensure the social factors impacting on the health of clients/ communities are addressed |

| **6. Communication with consumers** |
| --- |
| **Impact area and performance indicators** |
| **6.1. Communication principles/standards** |
| 6.1.1. We tailor our written and verbal communication to the specific needs of our target groups (e.g. culture, age, gender, sexuality, cognitive abilities etc.) |
| 6.1.2. We use appropriate and respectful terminology in all communication with consumers (e.g. when referring to specific cultural groups, gender and sexually diverse people etc.) |
| **6.2. Providing health information** |
| 6.2.1. Our staff routinely assess the learning needs, capacity and preferences of clients |
| 6.2.2. Our practitioners confirm that clients understand the information they have been provided |
| **6.3. Using media and technology** |
| 6.3.1. Our organisation is aware of, understands and effectively uses ethnic media |
| 6.3.5. We deliver social marketing and communication campaigns that are sustained over an adequate period of time |
| **6.4. Providing health education programs** |
| 6.4.1. We deliver health education and promotion initiatives that aim to build the health literacy of the community |
| 6.4.3. We work with community champions and mentors to provide peer education |

| **7. Workforce** |
| --- |
| **Impact area and performance indicators** |
| **7.1. Recruiting an appropriate workforce** |
| 7.1.1. Our organisation has established a set of health literacy competencies required by staff |
| 7.2.4. Our induction and orientation processes for new staff include information on health literacy |
| **7.2. Providing supportive working environments** |
| 7.2.1. Our staff are supported to provide flexible and consumer-centred care |
| 7.2.3. Staff are encouraged and supported to accurately document/record the (number and type) of services provided |
| **7.3. Providing practice tools and resources** |
| 7.3.5. Staff are provided with instruction guides on how to communicate effectively with clients (e.g. developing accessible reading materials) |
| 7.3.6. Staff are provided with the range of resources (e.g. voice recordings, educational materials, displays) they require to address the needs of clients |
| **7.4. Providing ongoing professional development** |
| 7.4.2. Our organisation regularly assesses the knowledge, skills and competencies of staff in relation to health literacy |
| 7.4.3. Our staff are supported to develop knowledge and skills relevant to health literacy |

## Priority Setting Tool

| **Date** |  |
| --- | --- |
| **Facilitator** |  |
| **Note Taker** |  |
| **Participants** |  |

| **Purpose** | The purpose of this tool is to identify aspects of your systems, processes and practices that require improvement, and determine priorities for implementing health literacy improvement activities. |
| --- | --- |
| **Process** | Firstly, have a brief reflection and discussion about your organisation’s strengths and weaknesses, using the questions below as a guide.  Secondly, identify and record the actions required to improve your organisation’s performance in each of the seven health literacy responsiveness domains.  Thirdly, rate the priority level of each action according to the following criteria:   1. This requires immediate action, as this is very likely to have significant impact on our overall performance, and prevents us from making improvements in many other areas. 2. This requires action, as it may have a significant impact on our overall performance and prevents us from making improvements in some other areas. 3. This requires gradual action, as it is not likely to have a significant impact on our overall performance and does not prevent us from making improvements in other areas.   Finally, indicate the resourcing required according to the following criteria:   1. Can be achieved with existing resources 2. Requires additional staff resources 3. Requires additional financial resources 4. Requires additional staff and financial resources |

| **Questions** | 1. What do we currently do well to support the health literacy needs of consumers/the community?  2. What could we do better to support the health literacy needs of consumers/the community?  3. What system/process/practice improvements need to occur within the organisation to strengthen our responsiveness to the health literacy needs of consumers/the community?  4. Do we currently have the available expertise, capacity and system capability to implement the required improvements? |
| --- | --- |
| **Top five strengths** | 1.  2.  3.  4.  5. |
| **Top five weaknesses** | 1.  2.  3.  4.  5. |

| **1. Policy and funding mandate** | | |
| --- | --- | --- |
| **Actions required to improve our performance in this area** | **Priority Level** | **Resources required** |
|  |  |  |
|  |  |  |
|  |  |  |
|  |  |  |
|  |  |  |
|  |  |  |
| **2. Leadership and culture** | | |
| **Actions required to improve our performance in this area** | **Priority Level** | **Resources required** |
|  |  |  |
|  |  |  |
|  |  |  |
|  |  |  |
|  |  |  |
|  |  |  |
|  |  |  |
|  |  |  |
| **3. Systems, processes and policies** | | |
| **Actions required to improve our performance in this area** | **Priority Level** | **Resources required** |
|  |  |  |
|  |  |  |
|  |  |  |
|  |  |  |
|  |  |  |
|  |  |  |
|  |  |  |
| **4. Access to programs and services** | | |
| **Actions required to improve our performance in this area** | **Priority Level** | **Resources required** |
|  |  |  |
|  |  |  |
|  |  |  |
|  |  |  |
|  |  |  |
|  |  |  |
|  |  |  |
|  |  |  |
|  |  |  |
| **5. Community engagement and partnerships** | | |
| **Actions required to improve our performance in this area** | **Priority Level** | **Resources required** |
|  |  |  |
|  |  |  |
|  |  |  |
|  |  |  |
|  |  |  |
|  |  |  |
|  |  |  |
|  |  |  |
|  |  |  |
| **6. Communication with consumers** | | |
| **Actions required to improve our performance in this area** | **Priority Level** | **Resources required** |
|  |  |  |
|  |  |  |
|  |  |  |
|  |  |  |
|  |  |  |
|  |  |  |
|  |  |  |
|  |  |  |
|  |  |  |
| **7. Workforce** | | |
| **Actions required to improve our performance in this area** | **Priority Level** | **Resources required** |
|  |  |  |
|  |  |  |
|  |  |  |
|  |  |  |
|  |  |  |
|  |  |  |
|  |  |  |
|  |  |  |
|  |  |  |

## Health Literacy Action Plan Template

| **Contact person:** |  |
| --- | --- |
| **Development date:** |  |
| **Review date:** |  |

| **Impact Area: e.g. Leadership and culture** | | | | |
| --- | --- | --- | --- | --- |
| **Improvement priorities** | **Strategies/Actions** | **Timeframes** | **Person/team responsible** | **Monitoring mechanism & measures** |
|  |  |  |  |  |
|  |  |  |  |  |
|  |  |  |  |  |
|  |  |  |  |  |

| **Impact Area:** | | | | |
| --- | --- | --- | --- | --- |
| **Priorities** | **Strategies/Actions** | **Timeframes** | **Person/team responsible** | **Monitoring mechanism & measures** |
|  |  |  |  |  |
|  |  |  |  |  |
|  |  |  |  |  |
|  |  |  |  |  |
|  |  |  |  |  |

# References

Batterham, R. W., Buchbinder, R., Beauchamp, A., Dodson, S., Elsworth, G. R. & Osborne, R. H. 2014, 'The OPtimising HEalth LIteracy (Ophelia) process: study protocol for using health literacy profiling and community engagement to create and implement health reform', *BMC Public Health,* vol. 14, no. pp.

Beauchamp, A., Buchbinder, R., Dodson, S., Batterham, R. W., Elsworth, G. R., McPhee, C., Sparkes, L., Hawkins, M. & Osborne, R. H. 2015, 'Distribution of health literacy strengths and weaknesses across socio-demographic groups: a cross-sectional survey using the Health Literacy Questionnaire (HLQ)', *BMC Public Health,* vol. 15, no. pp. 678.

Dodson, S., Beauchamp, A., Batterham, R. & Osborne, R. 2014, 'Ophelia Toolkit: A step-by-step guide for identifying and responding to health literacy needs within local communities', retrieved 4 May, <https://ophelia.net.au/>.

Ishikawa, H. & Yano, E. 2008, 'Patient health literacy and participation in the health-care process', *Health Expect,* vol. 11, no. 2, pp. 113-22.

Parker, R., Ratzan, S. & Lurle, N. 2003, 'Health literacy: A policy challenge for advancing high-quality health care', *Health Affairs,* vol. 22, no. 4, pp. 147-153.

Sorensen, K., Van den Broucke, S., Fullam, J., Doyle, G., Pelikan, J., Slonska, Z. & Brand, H. 2012, 'Health literacy in public health: A systematic review and integration of definitions and models ', *BMC Public Health,* vol. 12, no. 80, retrieved 17 April 2013, <[http://www.biomedcentral.com/1471-2458/12/80%3E](http://www.biomedcentral.com/1471-2458/12/80%3e).

Wolf, M. S., Gazmararian, J. A. & Baker, D. W. 2005, 'Health literacy and functional health status among older adults', *Arch Intern Med,* vol. 165, no. 17, pp. 1946-52.
