# Supplementary material for: Development of the Organisational Health Literacy Responsiveness (Org-HLR) self-assessment tool and process
Source: BMC Health Serv Res. 2018 Sep 6;18:694. doi: 10.1186/s12913-018-3499-6 (PMC6128002; doi:10.1186/s12913-018-3499-6)
Supplement: Supplementary file 3 — Org-HLR Self-Rating Tool (Truncated Version). (DOCX 42 kb) [file 12913_2018_3499_MOESM3_ESM.docx]

**Additional File 3:**

**Org-HLR Self-Rating Tool (Truncated Version)**

| **1. Policy and funding mandate** |
| --- |
| **Performance indicators** |
| 1.1. There are policy frameworks available to guide health literacy work within our organisation |
| 1.4. We are provided with incentives to drive health literacy responsive practice |

| **2. Leadership and culture** |
| --- |
| **Impact area and performance indicators** |
| **2.1. Financial management** |
| 2.1.1. Our organisation allocates adequate financial resources to appropriate service and program delivery |
| 2.1.3. Health literacy improvement activities are resourced over the long term |
| **2.2. Leadership and commitment** |
| 2.2.1. Our organisation has a vision and mission that explicitly expresses our commitment to equity, diversity and consumer-centred care |
| 2.2.4. There are health literacy champions within our organisation who advocate for positive change and improvements |
| **2.3. Health literacy is an organisational priority** |
| 2.3.2. Our organisation has clearly defined health literacy goals and objectives |
| 2.3.3. Health literacy is included in our strategic plan/business/operational plans |
| **2.4. Equity and diversity focused** |
| 2.4.1. Our organisation celebrates diversity and promotes acceptance and inclusion of people from all diverse groups |
| 2.4.4. Equity and diversity principles are embedded into organisational plans and policies |
| **2.5. Consumer-centred philosophy** |
| 2.5.1. Health literacy is viewed as a an individual and community asset and right |
| 2.5.2. There is a commitment to delivering consumer-centred care at all levels of our organisation |

| **3. Systems, processes and policies** |
| --- |
| **Impact area and performance indicators** |
| **3.1. Undertaking data collection and community needs identification** |
| 3.1.3. Comprehensive community needs assessments are regularly and systematically undertaken to develop a knowledge of community demographics, determine barriers to access and inform service and program |
| 3.1.9. Data are disaggregated according to gender, culture and language |
| **3.2. Undertaking performance monitoring and evaluation** |
| 3.2.1. A set of health literacy standards have been established within our organisation |
| 3.2.3. There is a process in place for monitoring, evaluating and reporting on the quality of our health literacy practice |
| **3.3. Undertaking service planning and quality improvement** |
| 3.3.1. We have a systematic process in place for undertaking service and program planning |
| 3.3.5 There is a mechanism in place for ensuring the outcomes of quality improvement activities are documented, reported and incorporated into future planning processes |
| **3.4. Communication systems and processes** |
| 3.4.4. Recall and reminder systems are in place to support appointment confirmation and post-appointment follow up |
| 3.4.6. There is a system and process in place for developing and reviewing all communication materials to ensure they meet health literacy standards |
| **3.5. Internal policies and procedures** |
| 3.5.1. We have policies and procedures in place to support effective engagement with clients and the community |
| 3.5.5. Relevant staff are involved in the development and review of policies and procedures |

| **4. Access to programs and services** |
| --- |
| **Impact area and performance indicators** |
| **4.1. Providing an appropriate service environment** |
| 4.1.1. Our buildings and venues/facilities are accessible (e.g. affordable parking, ramp access, and close to public transport) |
| 4.1.2. Our services provide a welcoming and supportive environment for diverse and vulnerable groups (including for Aboriginal people, CALD communities, GLBTIQ communities etc.) |
| **4.2. Supporting initial entry and ongoing access to services and programs** |
| 4.2.1. We have clear access and referral pathways in place |
| 4.2.8. Clients are provided with resources that support them to navigate their way through the health system |
| **4.3. Providing outreach services** |
| 4.3.1. We utilise a range of service delivery models to ensure we engage people and communities who are unable to attend services in person (e.g. outreach services, out of ours services, mobile and online) |
| 4.3.3. We deliver community based events to provide information and services to people not engaged in services (e.g. screening and health checks, immunisation) |

| **5. Community engagement and partnerships** |
| --- |
| **Impact area and performance indicators** |
| **5.1. Undertaking community consultation and enabling consumer participation** |
| 5.1.1. Our organisation consults with the community to develop an understanding of their health and health literacy needs |
| 5.1.5. We engage clients and communities in all aspects of service and program planning |
| **5.2. Partnerships with other organisations** |
| 5.2.1. Our organisation works collaboratively with service partners to co-design services, programs, materials and referral pathways |
| 5.2.4. Our organisation engages with services outside the health sector, such as housing, education and employment, to ensure the social factors impacting on the health of clients/ communities are addressed |

| **6. Communication with consumers** |
| --- |
| **Impact area and performance indicators** |
| **6.1. Communication principles/standards** |
| 6.1.1. We tailor our written and verbal communication to the specific needs of our target groups (e.g. culture, age, gender, sexuality, cognitive abilities etc.) |
| 6.1.2. We use appropriate and respectful terminology in all communication with consumers (e.g. when referring to specific cultural groups, gender and sexually diverse people etc.) |
| **6.2. Providing health information** |
| 6.2.1. Our staff routinely assess the learning needs, capacity and preferences of clients |
| 6.2.2. Our practitioners confirm that clients understand the information they have been provided |
| **6.3. Using media and technology** |
| 6.3.1. Our organisation is aware of, understands and effectively uses ethnic media |
| 6.3.5. We deliver social marketing and communication campaigns that are sustained over an adequate period of time |
| **6.4. Providing health education programs** |
| 6.4.1. We deliver health education and promotion initiatives that aim to build the health literacy of the community |
| 6.4.3. We work with community champions and mentors to provide peer education |

| **7. Workforce** |
| --- |
| **Impact area and performance indicators** |
| **7.1. Recruiting an appropriate workforce** |
| 7.1.1. Our organisation has established a set of health literacy competencies required by staff |
| 7.2.4. Our induction and orientation processes for new staff include information on health literacy |
| **7.2. Providing supportive working environments** |
| 7.2.1. Our staff are supported to provide flexible and consumer-centred care |
| 7.2.3. Staff are encouraged and supported to accurately document/record the (number and type) of services provided |
| **7.3. Providing practice tools and resources** |
| 7.3.5. Staff are provided with instruction guides on how to communicate effectively with clients (e.g. developing accessible reading materials) |
| 7.3.6. Staff are provided with the range of resources (e.g. voice recordings, educational materials, displays) they require to address the needs of clients |
| **7.4. Providing ongoing professional development** |
| 7.4.2. Our organisation regularly assesses the knowledge, skills and competencies of staff in relation to health literacy |
| 7.4.3. Our staff are supported to develop knowledge and skills relevant to health literacy |
